# Glucose Concentration Measurement in Human Blood Plasma Solutions with Microwave Sensors

**DOI:** 10.3390/s19173779

**Published:** 2019-08-31

**Authors:** Carlos G. Juan, Enrique Bronchalo, Benjamin Potelon, Cédric Quendo, José M. Sabater-Navarro

**Affiliations:** 1 Department of Systems Engineering and Automation, Miguel Hernández University, 03202 Elche, Spain; 2 Department of Communications Engineering, Miguel Hernández University, 03202 Elche, Spain; 3 Laboratoire des Sciences et Techniques de l’Information de la Communication et de la Connaissance, University of Brest, 29200 Brest, France

**Keywords:** dielectric losses, glucose sensor, human blood plasma, microwave resonator, multi-component study, permittivity, quality factor

## Abstract

Three microwave sensors are used to track the glucose level of different human blood plasma solutions. In this paper, the sensors are evaluated as glucose trackers in a context close to real human blood. Different plasma solutions sets were prepared from a human blood sample at several added glucose concentrations up to 10 wt%, adding also ascorbic acid and lactic acid at different concentrations. The experimental results for the different sensors/solutions combinations are presented in this work. The sensors show good performance and linearity as glucose level retrievers, although the sensitivities change as the rest of components vary. Different sensor behaviors depending upon the concentrations of glucose and other components are identified and characterized. The results obtained in terms of sensitivity are coherent with previous works, highlighting the contribution of glucose to the dielectric losses of the solution. The results are also consistent with the frequency evolution of the electromagnetic signature of glucose found in the literature, and are helpful for selecting frequency bands for sensing purposes and envisioning future approaches to the challenging measurement in real biological contexts. Discussion of the implications of the results and guidelines for further research and development of more accurate sensors is offered.

## 1. Introduction

In the last years, many efforts have been devoted to develop non-invasive blood glucose monitoring (NIBGM) technology. People with diabetes need to self-measure their blood glucose level (glycemia) several times every day, as a means to control the excursion of the glycemia out of the healthy range. The usual ways to make these measurements are invasive and painful, involving the pricking of the skin with a lancet in order to collect a drop of blood on a test strip [1]. Given the comfortless of the process, the frequency and effectivity of the measurements throughout the day is often reduced, thus yielding a poorer management of the disease.

Hence, NIBGM technology that is able to measure glycemia in a non-invasive, comfortable way would produce a remarkable enhancement in diabetes treatment. NIBGM technology could incrementally increase the number of measurements per day and provide a quicker detection of undesired events. It could even lead to continuous glucose monitoring (CGM), making it possible to detect almost instantaneously any glycemia change, and allow the individual or other devices to perform the right correction at the moment, noticeably enhancing the treatment of diabetes [2]. It would also reduce health costs, since they have been proven to be drastically lower when CGM is involved [3].

Research is actively being developed nowadays in this regard, considering different technologies and methods. For example, algorithms and models for predicting and tracking glycemia with a lower number of measurements have been developed and studied [4,5,6,7,8,9]. Despite their promising results, none of them has been fully successful for use by the general population, since they do not provide a direct glycemia measurement [10]. CGM based upon electrochemical means are also being studied [11,12,13,14,15,16], but the continuous need of disposable stuff and the errors they present [17] (mainly because of the inflammation of the skin in the surroundings of the sensor placing) suggest that alternative solutions should be explored [18,19]. Some other methods have been investigated, such as trying to measure glycemia from the individual’s breath [20,21], saliva [22], tears [23,24], or gingival crevicular fluid [25], although conclusive results have not been found yet. Research concerning optical techniques is also actively under development, chiefly based on mid-infrared and near-infrared spectroscopy [26,27,28,29], although some disturbing factors must be addressed before real application [30].

Notwithstanding these attempts, when non-invasiveness is required in the measurement process, sensors based upon radio frequency and microwave techniques are frequently involved due to their penetration capabilities (see [31]). The idea of tracking biological markers by the changes in the dielectric properties has already been successfully put in practice in several fields (two recent ones are [32,33], for example). In this sense, several works have characterized the variation of the dielectric permittivity of glucose-containing solutions when the glucose concentration changes [34,35,36]. This is a very interesting behavior, since a sensor that is able to track the dielectric variations of the medium should be suitable for tracking its glucose concentration. To characterize the whole medium and provide for application in a biomedical context, most of the biological tissues’ dielectric properties were measured and defined in [37], which is a reference work in this research field.

Therefore, based on these principles, some attempts for NIBGM have been studied concerning radio frequency and microwave sensors (for a couple of recent reviews, see [38,39]). The most common approach is to use microwave resonators as sensing devices, due to their sensitivity to the dielectric permittivity of the surrounding media. The application of different kinds of resonators with various configurations has been analyzed and assessed by several authors [40,41,42,43]. In the general case, these works have shown a promising behavior as glucose sensors when simple media are regarded (i.e., water–glucose solutions). However, they have not yet met the expectations of the diabetes community when real, biological media are concerned. This is mainly due to its complexity and the big number of variables taking place, often requiring sophisticated algorithms to analyze great deals of information to make the measurements converge in order to retrieve the glucose level [44]. Thus, they are still waiting for further research and development for application in real contexts.

In this work, we aim to analyze this problem in a controlled, semi-real biological medium, made out of solutions of human blood plasma, glucose, ascorbic acid, and lactic acid. Three microwave sensors are used to conduct a glucose concentration retrieval study in blood plasma solutions, aimed at the comparison and identification of sensor behaviors in a more realistic context. The lack of positive results in complex media, in addition to the good results found in simple media by the above-mentioned works, suggests that other components different from glucose may affect the changes in the dielectric permittivity. This work is intended to identify these other contributions by two more components that are present in blood (ascorbic and lactic acid), in order to provide for the further design of blood glucose concentration sensors. This work proves that it is possible to track different parameters in a single biological sample by means of a microwave sensor, and it studies how the sensitivity is affected. The results shown are useful to understand the behavior of the sensor in a broader sense, as well as to address the challenge of measuring in real biological contexts.

The paper is organized as follows. The description of the sensors used in this study, as well as its setup, in addition to the experimental procedure and the solutions employed are offered in Section 2. The obtained results are plotted and briefly commented on in Section 3. The discussion of the results, as well as their interpretation and implications, are shown in Section 4. Finally, the main conclusions and the most important aspects inferred from this study are gathered in Section 5.

## 2. Materials and Methods

### 2.1. Sensors Description

Three microwave sensors were used for measuring human plasma solutions [43]. The aim was to provide characterization and useful information about the behavior of the sensors when more realistic biological solutions are concerned. Hence, as sensors, three microstrip open-loop half-wave resonators were employed, hereinafter named R1, R2, and R3, having as resonant frequencies (without measuring any sample) 2 GHz, 5.7 GHz, and 8 GHz, respectively.

**Figure 1:**
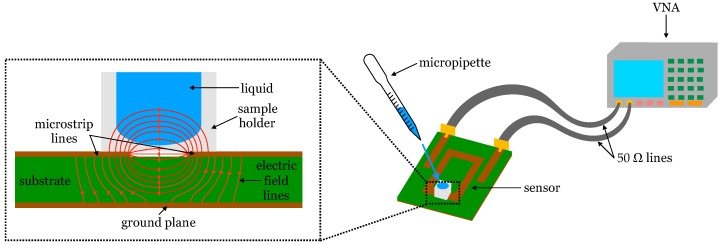
Illustration of the interaction of the electric field with the liquid under measurement, and of the measurement setup.

In the design, an open-loop geometry was chosen to exploit the high electric field region created between the open ends of the resonator for the first resonant mode. At resonance, there are voltage maxima of opposite signs in the open ends; therefore, this is the place where the electric interaction with the immediate upper space is the highest. For this reason, tracking the electrical response of each resonator when the sample is placed onto its open-end gap enables characterizing the sample, as illustrated in Figure 1. The convenience of having highly capacitive sensors (sensors exploiting high electric field zones) has recently been remarked by other authors [45]. In order to optimize the field–sample interaction, the gap length was chosen as a trade-off to avoid too weak intensity (long gap) and excessive field concentration in the interface between the substrate and the sample holder (short gap). This criterion led to gap lengths of 1600 µm for R1 and R2, and 1200 µm for R3.

A low-permittivity substrate (Taconic TLX-8, ε_r_ = 2.55, tan δ = 0.0017) was selected to reduce the influence of the substrate in the measurements. Within the available options, thick substrates were preferred to give a higher weight to the upper space than to the substrate in the energy distribution, having final substrate thicknesses of 1200 µm for R1 and 800 µm for R2 and R3. This avoids the fields being confined into the substrate, and hence they can be more affected by the media upon the circuit. Also, relatively high characteristic impedances were chosen in order to increase the field intensity at the open ends, since it is easy to show that the field intensity becomes greater as the characteristic impedance increases. To do it, narrow strip widths (within fabrication limitations) of 600 µm were selected, producing characteristic impedances of 117 Ω for R1 and 100 Ω for R2 and R3. Finally, the resonators were coupled to two 50 Ω I/O lines through coupled-line sections to conform a transmission configuration. The coupling strength was designed as a trade-off between too strong coupling (which would worsen the resolution of the unloaded *Q* factor of the resonators) and too weak coupling (which would lead to a too low resonant peak, unsuitable for measurement purposes), resulting in a coupling slot width of 500 µm for the three sensors.

**Figure 2:**
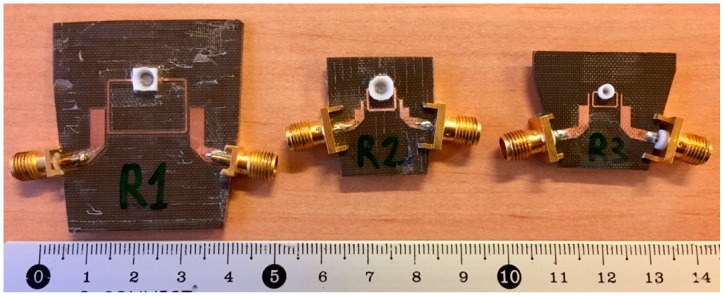
Sensors R1, R2, and R3 used in this study. The scale is in cm.

Special sample holders with an approximately hemispherical inner shape were designed and placed on the gap between the open ends of each resonator. These sample holders were glued onto the gap with a very thin layer (~50 µm) of epoxy resin (with roughly ε_r_ = 3.55, tan δ = 0.01). The chosen material was polytetrafluoroethylene (PTFE) because of its low permittivity, low losses, and low chemical reactivity, so that the influence of the sample holder in the measurements was minimum. A 25-µL inner volume PTFE sample holder was used for R1 and R2, and a 5-µL one was used for R3 (due to its smaller open-end gap), thus allowing for the characterization of very small samples. After gluing the sample holders and filling them with a reference sample of the human plasma used in this study, the resonant frequencies dropped to 1.92 GHz, 5.17 GHz, and 7.16 GHz, which were three frequency points within an interesting frequency range for biological sensing purposes, according to [37]. A picture of the sensors used in this study can be seen in Figure 2.

### 2.2. Experimental Procedure

The experimental study consisted of measuring the frequency response of the sensors R1, R2, and R3, having their sample holders filled with various blood plasma solutions with different concentrations of glucose and other substances (see Figures S1–S3 in Supplementary Materials section). For this purpose, O+ blood plasma from an unknown healthy donor (provided by Hospital General Universitario de Alicante, Alicante, Spain) was used. In this regard, the donors were informed, and the procedures were in accordance with the ethical standards of the Ethics Committee of the Hospital General Universitario de Alicante. The plasma was mixed with several additional substances, namely glucose, lactic acid (hereinafter labeled LA), and ascorbic acid (hereinafter labeled AA). Five sets of plasma solutions were prepared with different concentrations of AA and LA in each one. Each set consisted of five solutions with added glucose concentrations of 0%, 2.5%, 5%, 7.5%, and 10% in mass, thus yielding 25 solutions in aggregate. These concentrations are convenient to allow for comparison with other works and identify different behaviors of the sensors with biological solutions.

The concentrations of AA or LA in each set are shown in Table 1. The solutions were prepared by directly adding the solutes to the plasma samples, not mixing the plasma with previously diluted substances. The two values of AA and LA concentrations correspond to their respective low and high physiological limits [46].

It should be noted that the initial glucose content in the plasma sample was unknown, and the glucose concentrations expressed in this paper refer to the added glucose. However, the initial glucose concentration is supposed to be within the normal physiological range, and hence it may be deemed negligible in comparison with the added glucose amounts. In addition, all the solutions were prepared from the same plasma sample, and no variations in this parameter are expected. Also, as will be shown later, the measurements in this study are differential; the initial glucose concentration has no effect on the concentration raises. On the other hand, initial AA and LA concentrations were unknown as well, and they can be considered as concentration offsets, which can be roughly estimated as the mean of their normal physiological ranges (13 × 10^−6^ g/cm^3^ for AA, and 12.15 × 10^−5^ g/cm^3^ for LA).

Additionally, a sixth set was prepared by adding AA and LA to the plasma, both at half their high limit concentration, i.e., AA at 10 × 10^−6^ g/cm^3^ and LA at 9.9 × 10^−5^ g/cm^3^. This set was prepared to see the aggregate effects of the simultaneous presence of both acids, and it is labeled ‘Mix’ on what follows. 

A volume of 5 mL for each solution was prepared, of which samples of 5 and 25 µL were used in the measurements. The components employed were D(+)-glucose anhydrous from PanReac AppliChem (Castellar del Vallès, Spain) (ref. 131341), L-ascorbic acid from Sigma-Aldrich^®^ (Merck KGaA, Darmstadt, Germany) (ref. A7631), and L(+)-lactic acid from Scharlau (Sentmenat, Spain) (ref. AC1381).

All the solutions were characterized with the three microwave sensors (R1 to R3), thus performing 90 measurements. The frequency responses of the sensors (S-parameters) were measured with a vector network analyzer (VNA), previously calibrated with a Short–Open–Load–Through (SOLT) calibration kit. Special focus was placed on the transmission coefficient (S21). For each sensor, the frequency response with an empty holder was saved, frozen, and held in the VNA screen. For each solution, the sample holder was filled with a fixed volume (5 µL or 25 µL, depending on the sample holder) using a micropipette. After measuring the corresponding S-parameters, the sample holder was carefully cleaned with ethanol until the empty-case S-parameters frozen in the VNA screen were perfectly matched again, and hence the system was ready for a new measurement. The measurement setup can be seen in Figure 1. The sets were measured in the following order: P, AAL, AAH, LAL, LAH, and Mix. For each set, the measurements were performed in order from the lowest (0%) to the highest (10%) added glucose concentration. All the measurements were made at 25 °C room temperature. Deionized water and an unaltered sample of the blood plasma were measured at the beginning and at the end of the measurement session for each sensor, to account for repeatability. Identical frequency responses were obtained for all these control measurements.

## 3. Results

**Figure 3:**
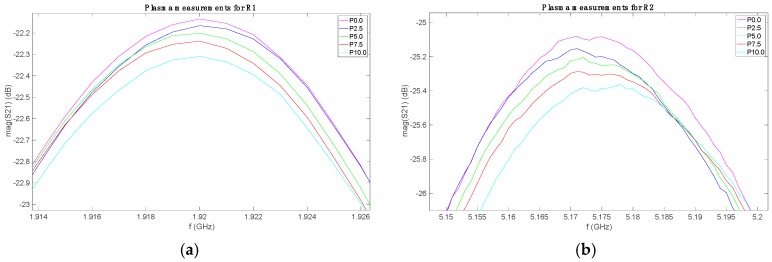

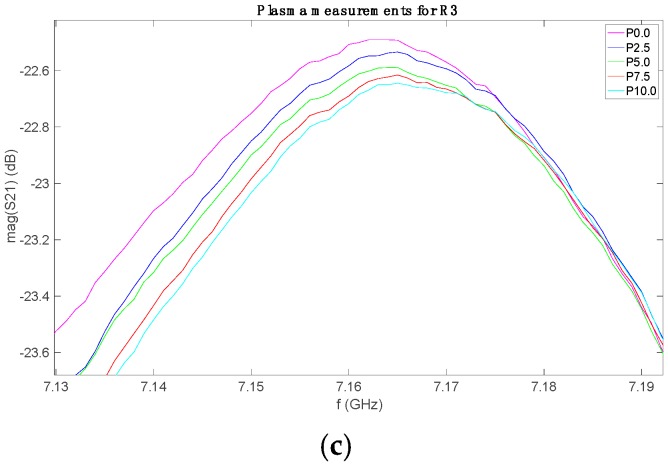
(**a**) Measured S21 parameter for plasma set with R1; (**b**) Measured S21 parameter for plasma set with R2; (**c**) Measured S21 parameter for plasma set with R3. R1, R2, and R3 are microstrip open-loop half-wave resonators with resonant frequencies of 2 GHz, 5.7 GHz, and 8 GHz, respectively.

The measurements for each set with each sensor were plotted together, in order to identify the possible behavior. As an example, Figure 3 shows the S21 parameters for the measurements of the plasma set (P) with the three sensors. The solutions are labeled as Px.x, where x.x indicates the added glucose mass percentage in the plasma solution. The rest of sets presented similar behaviors. All the data are freely available in [47].

As it can be seen, these graphs show the relationship between the measured frequency response and the sample glucose concentration. By paying attention to the plots in Figure 3a–c, one can note that the variations due to the glucose level are not seen in the resonant frequency (*f_r_*), but in the resonance 3-dB bandwidth (∆*f*_3dB_) and in the maximum amplitude of the S21 parameter (S21_max_, expressed in dB). The ∆*f*_3dB_ is the frequency range between the two frequencies for which the S21 magnitude falls 3 dB from S21_max_, at both sides of the resonance. These magnitudes are related to the resonator loaded (*Q*_L_) and unloaded (*Q*_u_) quality factors, which are given by:

(1) $$Q_{L}=\frac{f_{r}}{\Delta f_{3\mathrm{dB}}}$$

(2) $$Q_{u}=\frac{Q_{L}}{1-10^{\frac{S21_{\max}}{20}}}$$

While *Q*_L_ depends on the coupling strength between the resonator and the VNA ports, *Q*_u_ depends only on the resonator properties. Thus, on what follows, we will use *Q*_u_ as the magnitude determined by the resonance bandwidth.

The variations of the glucose concentration are expected to change the value of the dielectric permittivity, which is a complex, frequency-dependent parameter [31]:

(3) $$\varepsilon^{*}\left(f\right)=\varepsilon^{\prime }\left(f\right)-j\varepsilon^{\prime \prime }\left(f\right)$$

where *f* is the frequency. As a matter of fact, the variations of ε′ are expected to induce changes in the resonant frequency of the resonators, whilst the variations of ε″ are related to dielectric losses in the medium, and shall be noticed in the *Q*_u_ factor. The parameter S21_max_ depends on the resonator–VNA coupling and on *Q*_u_; therefore, it is indirectly affected by the losses. Thus, the very small variations in *f_r_*, along with the significant changes in S21_max_ and *Q*_u_ indicate that in the studied frequency range, the glucose concentration affects ε″ more than ε′. This is consistent with the data reported in [34] and with the results presented in [43] for water–glucose solutions in the present frequency range.

Therefore, a comprehensive analysis of the data was carried out in order to compute and plot these parameters, which can be seen in Tables S1–S3 in Supplementary Materials section. All the resonant frequencies were obtained and plotted, but no conclusive results were achieved, which was expected, since the variations were random and comparable to the VNA frequency resolution.

**Figure 4:**
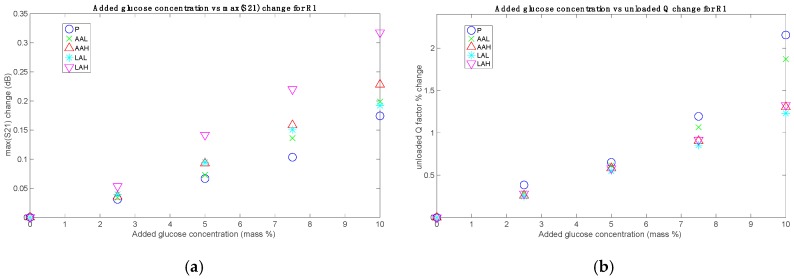
(**a**) Measurements with R1: absolute changes for S21_max_; (**b**) Measurements with R1: percentage changes for the resonator unloaded quality factor *Q*_u_.

**Figure 5:**
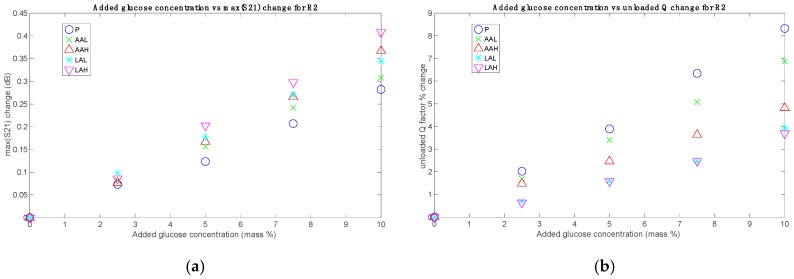
(**a**) Measurements with R2: absolute changes for S21_max_; (**b**) Measurements with R2: percentage changes for *Q*_u_.

**Figure 6:**
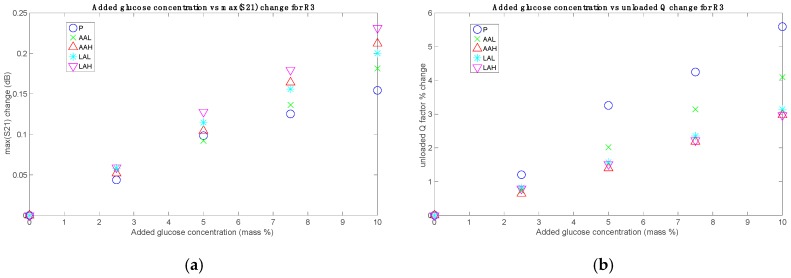
(**a**) Measurements with R3: absolute changes for S21_max_; (**b**) Measurements with R3: percentage changes for *Q*_u_.

The parameters S21_max_ and *Q*_u_ were computed for all the sets and added glucose concentrations. The results, grouped for each sensor, are shown in Figure 4, Figure 5 and Figure 6, without considering the Mix set. These figures represent for each set the absolute difference in the S21_max_ and the percentage change in the *Q*_u_, both with respect to their respective 0% added glucose measurement. These parameters are plotted against the added glucose concentration in mass percentage, thus allowing for the identification of glucose contribution to the changes of the measuring parameters. As it can be seen, clear relationships between the tracking parameters and the glucose level were obtained, which also showed a certain dependence on the acid content. While roughly the same tendencies of the responses concerning the added glucose concentration were obtained for all sets, the sensibility (in terms of the slope) seems to change for each one, having the greatest for the P set and the lowest for the LAH set for the unloaded *Q* factor, and the other way round for the S21_max_, in the general case.

**Figure 7:**
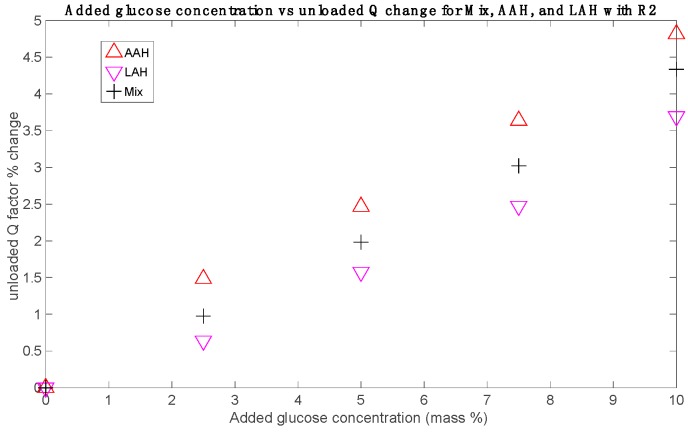
Results for *Q*_u_ measurements for the Mix, AAH, and LAH sets with R2.

Finally, the results for Mix set showed an intermediate behavior between AAH and LAH. This is a logical result, since the samples of this set have half the concentrations of the AAH and LAH samples. This also points out that their effects are additive. As an example, Figure 7 shows the unloaded *Q* factors percentage changes obtained for Mix set in comparison with those for AAH and LAH with the sensor R2. The rest of measurements for the Mix set resulted always in similar behaviors.

## 4. Discussion

In this section, the main focus will be on *Q*_u_ as sensing magnitude for the glucose concentration (*C*_g_). Although the parameter S21_max_ provides an alternative measurement of *C*_g_, they both are related, as it can be seen in Equations (1) and (2), and they therefore give essentially the same information regarding *C*_g_. Moreover, *Q*_u_ has the advantage of not depending upon the external coupling, since it is an intrinsic property of the resonator.

Within the added glucose concentration range of the solutions measured in this study (0–10% mass content), the variation of *Q*_u_ with respect to *C*_g_ is approximately linear for all the solution sets. The addition of other solutes alters the slope, but the behavior remains linear. In this discussion, we are not considering the possible chemical reactions between the added components and plasma, and it is assumed that the only component with a remarkably higher concentration than the physiological ones is glucose.

The *Q*_u_ sensitivities (*S_Q_*) obtained for all the sets with a simple least squares method can be seen in Table 2. Comparison with measurements with distilled water–glucose solutions (WG) is also presented. It is worthy to note that a glucose concentration increment leads to a *Q*_u_ decrement, but the corresponding negative sign is not included in *S_Q_* as the changes were computed in relation to percentage difference. The *Q*_u_ values obtained for the 0% added glucose measurement (denoted as *Q*_u0_) in each set are shown in Table 3. These are the values that are used as reference for computing the percentage differences.

In general, the linearity of the measurements results is good, with adjusted R^2^ values over 0.98 for the least squares approximation with sensors R2 and R3. Regarding R1, the behavior is less linear, with adjusted R^2^ values of 0.90 for the P set and 0.94 for the AAL set. The tracking parameter (*Q*_u_) presents in general a good correlation with the target magnitude (*C*_g_), as it can be inferred from the correlation coefficients (R) obtained for the three sensors when measuring all the solutions sets, as shown in Table 4. The correlation coefficients obtained in this work compare well with the ones obtained with WG solutions. This means that the measurement principle seems right, and the differences are not found in the linearity, but rather in the sensitivity. In all the sets, and for the three sensors, the sensitivity is lower than the one obtained for water–glucose solutions. This result can be explained by estimating the resonator unloaded quality factor considering the sample as the only loss factor, i.e., disregarding the ohmic or radiation losses in the microstrip line, as well as the substrate dielectric losses. With these assumptions, it is easy to express the *Q*_u_ sensitivity with respect to *C*_g_ as:

(4) $$S_{Q}=\frac{\Delta Q_{u}\left(\%\right)}{\Delta C_{g}\left(\%\right)}\sim 100\frac{Q_{u}}{Q_{u0}}\Delta \frac{1}{\varepsilon^{\prime \prime }}\Delta \frac{\Delta \varepsilon^{\prime \prime }}{\Delta C_{g}}$$

This expression clearly shows that an increase in the dielectric losses yields to a decrease in the sensitivity. In blood plasma, there are at least two additional loss factors in comparison to water for the same glucose concentration: a greater ionic conductivity, due to the presence of electrolytes, and a greater viscosity, associated with the presence of several organic molecules. A viscosity rise moves the frequency at which the ε″ is maximum, which is roughly 20 GHz for pure water [48], toward lower frequencies. This is due to the proportional relationship between the dielectric relaxation time and the viscosity [49]. For the frequencies considered in this study (within the 2–7 GHz range), the final effect results in dielectric losses increment. This effect can be seen in a clear manner in Figure 1 of [50] (p. 3). The losses associated to the ionic conductivity, which are greater for low frequencies, might also explain why the sensitivity of sensor R1 is lower (see Table 2).

The experimental values of *S_Q_* obtained for plasma are coherent with the values that can be expected from Equation (4). For our measurements, it can be assumed that the *Q*_u_*/Q*_u0_ ratio is slightly lower than 1 (see Table 2 and Table 3) and ε″∼20 (a usual value for water in the considered frequency range), whereas ∆ε″/∆*C*_g_ can be set from the references shown in Table 5 (some of these data were obtained from the original plots by means of a graphic data extraction software, and must be therefore considered as approximate):

Thus, having an average value for ∆ε″/∆*C*_g_∼0.25/wt% obtained from Table 5, and applying it to Equation (4) yields *S_Q_*∼1.2%/%. This estimation is comparable to the experimental values presented in Table 2 for plasma solutions. The differences, as explained before, can be due to the higher dielectric losses of plasma.

**Figure 8:**
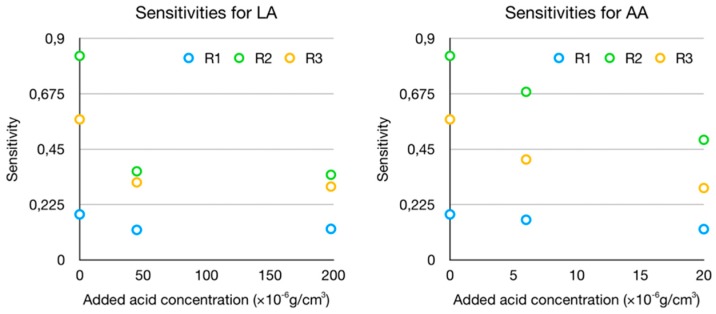
Sensitivities of the sensors to the added acids concentrations.

The sensitivities for the AA and LA sets are always lower than those for the P sets, as shown in Figure 8 (where the sensitivities of the P sets are the dots at 0 added acid concentration). Specifically, the sensitivities for AAL with respect to P decrease to 88.11%, 82.39%, and 71.45% for R1, R2, and R3, respectively, whereas those for LAL with respect to P decrease to 69.95%, 43.43%, and 55.17%. In this figure, due to the unknown prior concentrations of the acids, all the points could be displaced in the *x*-axis by a certain offset, with the behavior remaining unaltered. An approximation for this offset can be taken as the mean value for the physiological range of each acid.

Besides, the increase of AA or LA concentration leads in both cases to a decrease in the sensitivity, as it is clearly seen in Figure 8. This decrease is not linear, and it seems to be more related to a saturation effect; that is to say, the sensitivity seems to trend toward a limit value at high concentrations (at least within the physiological ranges). In the case of LA sets measured with R1, this saturation state seems to have been reached, and the small sensitivity increase from LAL to LAH might be due to instrumental errors. It should be noted that the sensitivity reduction regarding LA sets is only slightly greater than the sensitivity reduction regarding AA sets, even though the AA concentrations are one order of magnitude smaller. This could be due to a greater influence, in relative terms, of ascorbic acid because of its greater molecular size (six carbon atoms in the AA molecule, C_6_H_8_O_6_, for three in the LA molecule, C_3_H_6_O_3_). Concerning the results for the Mix set, the sensitivity is quite approximately the mean of the AAH and LAH sensitivities (see Figure 7).

To the best of our knowledge, very few data are available concerning the dielectric properties of these acids. The relative permittivity of water–LA solutions was studied in [53]. At 2.45 GHz and ∼25 °C, the effective relative permittivity of a solution at 14.6% in mass was shown to be ε_r,eff_^*^∼9–j5.5. The relative permittivity of deionized water at the same temperature and frequency is ∼77–j10 [48]. Therefore, the relative dielectric permittivity of LA can be estimated by means of the Maxwell–Garnett formula:

(5) $$\varepsilon_{r,\mathrm{eff}}^{*}=\varepsilon_{1}\frac{\varepsilon_{2}\left(1+2v\right)+2\varepsilon_{1}\left(1+v\right)}{\varepsilon_{2}\left(1-v\right)+\varepsilon_{1}\left(2+v\right)}$$

where *ε*_1_ and *ε*_2_ are the relative permittivities of the solvent (water) and the solute (LA), respectively, and *v* is the volume fraction of the solvent. Approximating *v* as the mass fraction (which induces low error in aqueous solutions), the above-mentioned data can be used to solve Equation (5) for *ε*_2_, giving *ε*_2_ (LA) ≈ 1.18–j4.75. This estimation, specifically regarding the imaginary part of the relative permittivity, accounts for the noticeable contribution of LA to the overall dielectric losses of the solution, relative to its concentration. This is consistent with the data reported in Table 2.

Although our attention has been focused upon *Q*_u_ as a sensing magnitude for *C*_g_, the experimental results for the sensitivity of S21_max_ with respect to the added glucose concentration (ΔS21_max_/Δ*C*_g_) are shown in Table 6. Provided the existing relationship between *Q*_u_ and S21_max_ [see Equations (1) and (2)], it is easy to obtain the relationship between ΔS21_max_/Δ*C*_g_ and *S_Q_*. The theoretical estimations thereby calculated from the experimental values of *S_Q_* in Table 2 and Table 3 are similar to the experimental values in Table 6, except for small differences that can be put down to experimental errors.

For microwave resonators in the frequency range concerned in this work, the sensitivity reduction for complex solutions, such as in blood plasma, in comparison to that for pure water, shows the need for further research before application for future non-invasive sensors. New designs should be studied, aimed at maximizing the interaction of the electromagnetic fields with the sample and thereby gaining sensitivity. In this sense, the study of new options for placing the sample with strategic structures to amplify the field seems advisable. The results in this work also suggest broadening the study of the glucose influence in the dielectric behavior of plasma to other frequency bands.

## 5. Conclusions

The performance of three microwave sensors for glucose concentration has been analyzed when human blood plasma solutions are concerned. The assessment included, in addition to glucose, the use of ascorbic acid and lactic acid. The results have shown how the three sensors are able to track the glucose variations in all the considered situations, provided that the rest of the components in the solution are known. This entails a step forward toward the development of a NIBGM device in a real, complex environment, as this study allows identifying and characterizing the behavior of this kind of sensors when biological solutions are regarded, as well as when the concentrations of other components different from glucose are changing. 

The results have revealed a better performance in terms of the sensitivity for R2 and R3 than that for R1, thus pointing to higher frequencies as desirable for future designs. They have also underlined the importance of individual calibration (as it was pointed out by other authors [54]), as well as the need for multicomponent tracking. In this sense, the comprehensive modeling of the real environment of application is deemed as essential for the success of future NIBGM proposals. Due to these reasons, further research on new sensors based on the principles discussed in this work is advised, which should include different frequencies and measuring parameters, and should involve several technologies and physical principles. The information gathered from them all will serve to feed machine learning algorithms devoted to building complex, trustable models of the real environment in order to understand all the phenomena occurring from an electromagnetic point of view. Once such a device will be ready, and the algorithms will provide accurate models for each individual, the composition of the main parameters, including glucose level, should be retrievable from new measurements of the sensors. Research on real biological conditions, such as the ones presented in this paper, is essential for advancing toward these future systems.

As to future scope, new techniques to gain sensitivity will be investigated, based on the principles seen in this work, trying to maximize the interaction of the fields with the sample. The conclusions reached in this paper suggest involving higher frequencies for future attempts. In addition, it seems important to consider different measurement principles, frequencies, or devices benefiting from the different behaviors shown in this paper to gain selectivity and discern the glucose level from the measurements, regardless the rest of the components.

## Figures and Tables

**Table 1:** Solutions sets used in the study. AA: ascorbic acid, LA: lactic acid.

Label	Concentrations of AA or LA Added to Plasma
P	No additional components
AAL	AA at low limit (6 × 10^−6^ g/cm^3^)
AAH	AA at high limit (20 × 10^−6^ g/cm^3^)
LAL	LA at low limit (4.5 × 10^−5^ g/cm^3^)
LAH	LA at high limit (19.8 × 10^−5^ g/cm^3^)

**Table 2:** Sensitivities of the sensors for all sets regarding *Q*_u_. * Data from [43]. WG: water–glucose solution.

	*S_Q_* = ∆*Q*_u_/∆*C*_g_ (%/%)					
Sensor	WG *	P	AAL	AAH	LAL	LAH
R1	0.609	0.185	0.163	0.125	0.122	0.126
R2	0.978	0.829	0.683	0.488	0.360	0.346
R3	0.584	0.571	0.408	0.292	0.315	0.298

**Table 3:** *Q*_u0_ values obtained for the 0% added glucose measurement in each set. * Data from [43].

	*Q*_*u*0_					
Sensor	WG *	P	AAL	AAH	LAL	LAH
R1	76.454	70.531	70.346	70.296	70.001	69.909
R2	60.652	58.159	57.710	57.647	57.543	55.984
R3	72.682	64.992	64.987	64.963	64.967	64.965

**Table 4:** Correlation coefficients of the sensors for all sets regarding *Q*_u_. * Data from [43].

	Correlation Coefficient					
Sensor	WG *	P	AAL	AAH	LAL	LAH
R1	0.999	0.966	0.979	0.998	0.994	0.997
R2	0.997	0.999	0.999	0.999	0.991	0.997
R3	0.994	0.989	0.998	0.999	0.997	0.999

**Table 5:** Values of ∆ε″/∆*C*_g_ obtained from the scientific literature.

Reference	Medium	*f* (GHz)	∆ε″/∆*C*_g_ (/wt%)
[50]	water + glucose	2–3	0.19
[51]	water + glucose	5	0.25
[36]	water + glucose	6.5	0.5
[52]	pig blood	7.7	0.17

**Table 6:** Sensitivities of the sensors for all sets regarding S21_max_. * Data from [43].

	∆S21_max_/∆*C*_g_ (dB/%)					
Sensor	WG *	P	AAL	AAH	LAL	LAH
R1	0.047	0.017	0.020	0.023	0.019	0.032
R2	0.084	0.028	0.031	0.037	0.034	0.041
R3	0.048	0.015	0.018	0.021	0.020	0.023

## Supplementary Materials

The following are available online at https://www.mdpi.com/1424-8220/19/17/3779/s1, Figure S1: Measurement with R1, Figure S2: Measurement with R2, Figure S3: Measurement with R3, Table S1: results of the measurements with R1, Table S2: results of the measurements with R2, Table S3: results of the measurements with R3.

## Abbreviations

The following abbreviations are used in this manuscript:

CGM	Continuous Glucose Monitoring
NIBGM	Non-Invasive Blood Glucose Monitoring
PTFE	Polytetrafluoroethylene
SOLT	Short–Open–Load–Through
VNA	Vector Network Analyzer
WG	Water–Glucose
