# Supplementary material for: Glucose Concentration Measurement in Human Blood Plasma Solutions with Microwave Sensors
Source: Sensors (Basel). 2019 Aug 31;19(17):3779. doi: 10.3390/s19173779 (PMC6749577; doi:10.3390/s19173779)
Supplement: Supplementary file 1 [file sensors-19-03779-s001.zip › Table S3.docx]

| LIQUID | Fr exper | BW3dB  Results of the measurements with R3 | Q loaded | Q unloaded | Max S21 | Gluc. | % shift |
| --- | --- | --- | --- | --- | --- | --- | --- |
|  |  |  |  |  |  |  |  |
| Air | 7.5600e+009 | 7.974060e+007 | 94.807410 |  | -17.566348 | **-** |  |
| 0 | 7.1630e+009 | 1.191586e+008 | 60.113162 | 64.9923031 | -22.490371 | **0** | 0 |
| 2_5 | 7.1650e+009 | 1.205941e+008 | 59.414167 | 64.2103684 | -22.534155 | **2.5** | 1.203119 |
| 5 | 7.1650e+009 | 1.230930e+008 | 58.208034 | 62.8750402 | -22.588798 | **5** | 3.257713 |
| 7_5 | 7.1650e+009 | 1.243537e+008 | 57.625968 | 62.2308623 | -22.615723 | **7.5** | 4.248874 |
| 10 | 7.1650e+009 | 1.260730e+008 | 56.832116 | 61.3571796 | -22.644814 | **10** | 5.593160 |
|  |  |  |  |  |  |  |  |
| AAL0 | 7.1620e+009 | 1.193801e+008 | 60.104970 | 64.9872959 | -22.484034 | **0** | 0 |
| AAL2_5 | 7.1650e+009 | 1.200151e+008 | 59.700817 | 64.5151175 | -22.542566 | **2.5** | 0.726570 |
| AAL5 | 7.1640e+009 | 1.215265e+008 | 58.942147 | 63.6754443 | -22.576164 | **5** | 2.018628 |
| AAL7_5 | 7.1650e+009 | 1.232070e+008 | 58.289386 | 62.9445945 | -22.620386 | **7.5** | 3.143232 |
| AAL10 | 7.1640e+009 | 1.243150e+008 | 57.738132 | 62.3234959 | -22.665560 | **10** | 4.098955 |
|  |  |  |  |  |  |  |  |
| AAH0 | 7.1650e+009 | 1.192131e+008 | 60.094047 | 64.9626775 | -22.505142 | **0** | 0 |
| AAH2_5 | 7.1640e+009 | 1.199290e+008 | 59.735337 | 64.5437163 | -22.557105 | **2.5** | 0.644926 |
| AAH5 | 7.1650e+009 | 1.206633e+008 | 59.313222 | 64.0566248 | -22.609481 | **5** | 1.394728 |
| AAH7_5 | 7.1640e+009 | 1.216015e+008 | 58.871020 | 63.5441484 | -22.669357 | **7.5** | 2.183606 |
| AAH10 | 7.1650e+009 | 1.223537e+008 | 58.422768 | 63.0327302 | -22.717375 | **10** | 2.970856 |
|  |  |  |  |  |  |  |  |
| LAL0 | 7.1640e+009 | 1.193936e+008 | 60.102868 | 64.9673415 | -22.513185 | **0** | 0 |
| LAL2_5 | 7.1640e+009 | 1.201148e+008 | 59.642929 | 64.4353521 | -22.571381 | **2.5** | 0.818857 |
| LAL5 | 7.1650e+009 | 1.207433e+008 | 59.219970 | 63.945116 | -22.627843 | **5** | 1.573445 |
| LAL7_5 | 7.1640e+009 | 1.219822e+008 | 58.774673 | 63.4402584 | -22.669176 | **7.5** | 2.350540 |
| LAL10 | 7.1650e+009 | 1.228421e+008 | 58.321041 | 62.9252079 | -22.713472 | **10** | 3.143323 |
|  |  |  |  |  |  |  |  |
| LAH0 | 7.1630e+009 | 1.191920e+008 | 60.097297 | 64.9650154 | -22.507082 | **0** | 0 |
| LAH2_5 | 7.1630e+009 | 1.201120e+008 | 59.656363 | 64.4529724 | -22.566172 | **2.5** | 0.788183 |
| LAH5 | 7.1640e+009 | 1.208079e+008 | 59.255944 | 63.9798215 | -22.634888 | **5** | 1.516499 |
| LAH7_5 | 7.1650e+009 | 1.216487e+008 | 58.858005 | 63.5200746 | -22.686645 | **7.5** | 2.224183 |
| LAH10 | 7.1650e+009 | 1.223371e+008 | 58.439080 | 63.0382573 | -22.738481 | **10** | 2.965840 |
|  |  |  |  |  |  |  |  |
| MIX0 | 7.1640e+009 | 1.192028e+008 | 60.095720 | 64.9603445 | -22.511980 | **0** | 0 |
| MIX2_5 | 7.1650e+009 | 1.200643e+008 | 59.692356 | 64.4924785 | -22.565135 | **2.5** | 0.720233 |
| MIX5 | 7.1650e+009 | 1.207280e+008 | 59.280742 | 64.0173423 | -22.616618 | **5** | 1.451658 |
| MIX7_5 | 7.1650e+009 | 1.219188e+008 | 58.863857 | 63.5299219 | -22.680551 | **7.5** | 2.201994 |
| MIX10 | 7.1640e+009 | 1.223409e+008 | 58.435072 | 63.0397113 | -22.728372 | **10** | 2.956624 |
|  |  |  |  |  |  |  |  |
