# Supplementary material for: Glucose Concentration Measurement in Human Blood Plasma Solutions with Microwave Sensors
Source: Sensors (Basel). 2019 Aug 31;19(17):3779. doi: 10.3390/s19173779 (PMC6749577; doi:10.3390/s19173779)
Supplement: Supplementary file 1 [file sensors-19-03779-s001.zip › Table S2.docx]

| LIQUID | Fr exper | BW3dB  Results of the measurements with R2 | Q loaded | Q unloaded | Max S21 | Gluc. | % shift |
| --- | --- | --- | --- | --- | --- | --- | --- |
|  |  |  |  |  |  |  |  |
| Air | 5.7350e+009 | 6.684726e+007 | 85.792592 |  | -18.699328 | **-** |  |
| P0 | 5.1710e+009 | 9.584223e+007 | 54.974120 | 58.1589406 | -25.079984 | **0** | 0 |
| P2_5 | 5.1710e+009 | 9.777334e+007 | 53.887624 | 56.9805996 | -25.153221 | **2.5** | 2.026070 |
| P5 | 5.1720e+009 | 9.969375e+007 | 52.878878 | 55.8943603 | -25.203419 | **5** | 3.893778 |
| P7_5 | 5.1710e+009 | 1.022738e+008 | 51.560361 | 54.4693923 | -25.287137 | **7.5** | 6.343906 |
| P10 | 5.1780e+009 | 1.045954e+008 | 50.495505 | 53.3174014 | -25.362102 | **10** | 8.324669 |
|  |  |  |  |  |  |  |  |
| AAL0 | 5.1680e+009 | 9.476228e+007 | 54.536468 | 57.7101987 | -25.193650 | **0** | 0 |
| AAL2_5 | 5.1680e+009 | 9.633929e+007 | 53.643744 | 56.7347462 | -25.274996 | **2.5** | 1.690260 |
| AAL5 | 5.1690e+009 | 9.803802e+007 | 52.734643 | 55.7455508 | -25.350255 | **5** | 3.404334 |
| AAL7_5 | 5.1700e+009 | 9.970791e+007 | 51.851455 | 54.7813694 | -25.435559 | **7.5** | 5.075064 |
| AAL10 | 5.1700e+009 | 1.004983e+008 | 50.892673 | 53.7453149 | -25.501867 | **10** | 6.870335 |
|  |  |  |  |  |  |  |  |
| AAH0 | 5.1680e+009 | 9.490393e+007 | 54.465606 | 57.6465031 | -25.164467 | **0** | 0 |
| AAH2_5 | 5.1690e+009 | 9.628274e+007 | 53.685634 | 56.7918649 | -25.241048 | **2.5** | 1.482550 |
| AAH5 | 5.1700e+009 | 9.755779e+007 | 53.181416 | 56.2248246 | -25.331357 | **5** | 2.466201 |
| AAH7_5 | 5.1700e+009 | 9.808456e+007 | 52.577655 | 55.5506957 | -25.429773 | **7.5** | 3.635619 |
| AAH10 | 5.1700e+009 | 9.945122e+007 | 51.975229 | 54.8710870 | -25.531326 | **10** | 4.814544 |
|  |  |  |  |  |  |  |  |
| LAL0 | 5.1700e+009 | 9.515025e+007 | 54.335116 | 57.5434541 | -25.074316 | **0** | 0 |
| LAL2_5 | 5.1690e+009 | 9.573029e+007 | 53.995448 | 57.1458636 | -25.172339 | **2.5** | 0.690940 |
| LAL5 | 5.1690e+009 | 9.654635e+007 | 53.539052 | 56.6327891 | -25.251691 | **5** | 1.582569 |
| LAL7_5 | 5.1710e+009 | 9.740380e+007 | 53.088278 | 56.1215271 | -25.344428 | **7.5** | 2.471049 |
| LAL10 | 5.1720e+009 | 9.882026e+007 | 52.315417 | 55.2776666 | -25.418561 | **10** | 3.937524 |
|  |  |  |  |  |  |  |  |
| LAH0 | 5.1700e+009 | 9.754565e+007 | 52.864322 | 55.9836186 | -25.080086 | **0** | 0 |
| LAH2_5 | 5.1710e+009 | 9.807483e+007 | 52.557447 | 55.6263291 | -25.166004 | **2.5** | 0.638204 |
| LAH5 | 5.1710e+009 | 9.928719e+007 | 52.102226 | 55.1016302 | -25.282589 | **5** | 1.575440 |
| LAH7_5 | 5.1700e+009 | 1.000970e+008 | 51.659872 | 54.5994216 | -25.378145 | **7.5** | 2.472504 |
| LAH10 | 5.1720e+009 | 1.023585e+008 | 51.050321 | 53.916518 | -25.488316 | **10** | 3.692331 |
|  |  |  |  |  |  |  |  |
| MIX0 | 5.1690e+009 | 9.621623e+007 | 53.702962 | 56.8575077 | -25.117020 | **0** | 0 |
| MIX2_5 | 5.1710e+009 | 9.701642e+007 | 53.211245 | 56.3046159 | -25.202240 | **2.5** | 0.972417 |
| MIX5 | 5.1720e+009 | 9.811060e+007 | 52.705826 | 55.7321653 | -25.303766 | **5** | 1.979233 |
| MIX7_5 | 5.1750e+009 | 9.923564e+007 | 52.178174 | 55.1398686 | -25.398509 | **7.5** | 3.020954 |
| MIX10 | 5.1710e+009 | 1.070627e+008 | 51.500802 | 54.3928466 | -25.506736 | **10** | 4.334803 |
|  |  |  |  |  |  |  |  |
