# Supplementary material for: Glucose Concentration Measurement in Human Blood Plasma Solutions with Microwave Sensors
Source: Sensors (Basel). 2019 Aug 31;19(17):3779. doi: 10.3390/s19173779 (PMC6749577; doi:10.3390/s19173779)
Supplement: Supplementary file 1 [file sensors-19-03779-s001.zip › Table S1.docx]

| LIQUID | Fr exper | BW3dB  Results of the measurements with R1 | Q loaded | Q unloaded | Max S21 | Gluc. | % shift |
| --- | --- | --- | --- | --- | --- | --- | --- |
|  |  |  |  |  |  |  |  |
| Air | 2.0300e+009 | 2.105911e+007 | 96.395317 |  | -16.857199 | **-** |  |
| P0 | 1.9200e+009 | 2.948973e+007 | 65.107410 | 70.6314798 | -22.134783 | **0** | 0 |
| P2_5 | 1.9200e+009 | 2.959462e+007 | 64.876649 | 70.3599361 | -22.165689 | **2.5** | 0.384451 |
| P5 | 1.9200e+009 | 2.966364e+007 | 64.725706 | 70.1719017 | -22.201400 | **5** | 0.650670 |
| P7_5 | 1.9200e+009 | 2.981624e+007 | 64.394430 | 69.7879405 | -22.238177 | **7.5** | 1.194282 |
| P10 | 1.9200e+009 | 3.010424e+007 | 63.811597 | 69.1092252 | -22.309091 | **10** | 2.155207 |
|  |  |  |  |  |  |  |  |
| AAL0 | 1.9200e+009 | 2.960070e+007 | 64.863326 | 70.3463202 | -22.164472 | **0** | 0 |
| AAL2_5 | 1.9200e+009 | 2.967546e+007 | 64.699928 | 70.145925 | -22.198501 | **2.5** | 0.284869 |
| AAL5 | 1.9200e+009 | 2.976221e+007 | 64.511335 | 69.9152231 | -22.237308 | **5** | 0.612821 |
| AAL7_5 | 1.9200e+009 | 2.988170e+007 | 64.256178 | 69.5963789 | -22.300581 | **7.5** | 1.066070 |
| AAL10 | 1.9200e+009 | 3.006410e+007 | 63.771818 | 69.0305263 | -22.363242 | **10** | 1.870452 |
|  |  |  |  |  |  |  |  |
| AAH0 | 1.9200e+009 | 2.961836e+007 | 64.824645 | 70.2955324 | -22.177399 | **0** | 0 |
| AAH2_5 | 1.9200e+009 | 2.968523e+007 | 64.678612 | 70.1128244 | -22.213217 | **2.5** | 0.259914 |
| AAH5 | 1.9200e+009 | 2.973019e+007 | 64.504629 | 69.8857282 | -22.270350 | **5** | 0.582973 |
| AAH7_5 | 1.9200e+009 | 2.984324e+007 | 64.336164 | 69.659635 | -22.335726 | **7.5** | 0.904606 |
| AAH10 | 1.9210e+009 | 2.996786e+007 | 64.118811 | 69.3782699 | -22.405648 | **10** | 1.304866 |
|  |  |  |  |  |  |  |  |
| LAL0 | 1.9200e+009 | 2.973498+007 | 64.570405 | 70.0014132 | -22.204527 | **0** | 0 |
| LAL2_5 | 1.9200e+009 | 2.985052e+007 | 64.434411 | 69.8273648 | -22.243979 | **2.5** | 0.248636 |
| LAL5 | 1.9200e+009 | 2.989315e+007 | 64.274199 | 69.6169561 | -22.298992 | **5** | 0.549213 |
| LAL7_5 | 1.9200e+009 | 2.995590e+007 | 64.111458 | 69.4036186 | -22.354982 | **7.5** | 0.853975 |
| LAL10 | 1.9210e+009 | 3.016650e+007 | 63.892867 | 69.1396072 | -22.396747 | **10** | 1.231127 |
|  |  |  |  |  |  |  |  |
| LAH0 | 1.9220e+009 | 2.976999e+007 | 64.461652 | 69.9087865 | -22.179689 | **0** | 0 |
| LAH2_5 | 1.9210e+009 | 2.983603e+007 | 64.314170 | 69.7127406 | -22.233118 | **2.5** | 0.280431 |
| LAH5 | 1.9210e+009 | 2.987944e+007 | 64.175202 | 69.5032353 | -22.321250 | **5** | 0.580115 |
| LAH7_5 | 1.9220e+009 | 2.998291e+007 | 64.005620 | 69.2678639 | -22.399747 | **7.5** | 0.916798 |
| LAH10 | 1.9220e+009 | 3.008686e+007 | 63.798458 | 68.9804535 | -22.497163 | **10** | 1.327920 |
|  |  |  |  |  |  |  |  |
| MIX0 | 1.9210e+009 | 3.045572e+007 | 63.075169 | 68.3233879 | -22.291149 | **0** | 0 |
| MIX2_5 | 1.9210e+009 | 3.053006e+007 | 62.921584 | 68.1381381 | -22.320131 | **2.5** | 0.271137 |
| MIX5 | 1.9210e+009 | 3.058499e+007 | 62.750860 | 67.8926157 | -22.414222 | **5** | 0.630490 |
| MIX7_5 | 1.9220e+009 | 3.070165e+007 | 62.597330 | 67.6840228 | -22.481013 | **7.5** | 0.935792 |
| MIX10 | 1.9220e+009 | 3.082796e+007 | 62.375207 | 67.3931236 | -22.561843 | **10** | 1.361560 |
|  |  |  |  |  |  |  |  |
