# Supplementary figures and images for: Glucose Concentration Measurement in Human Blood Plasma Solutions with Microwave Sensors
Source: Sensors (Basel). 2019 Aug 31;19(17):3779. doi: 10.3390/s19173779 (PMC6749577; doi:10.3390/s19173779)

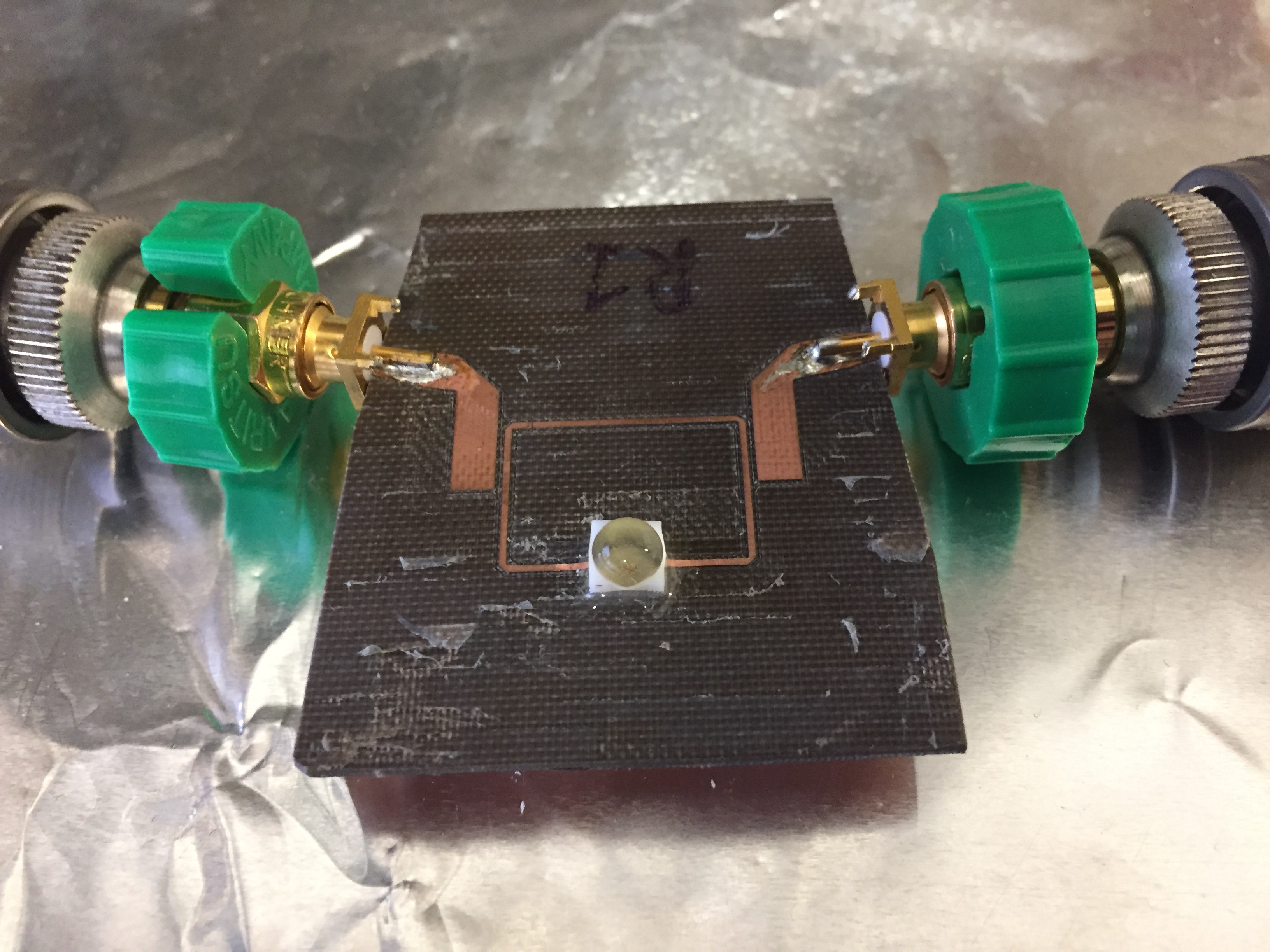

Supplement: Supplementary file 1 [file sensors-19-03779-s001.zip › Figure S1.JPG]

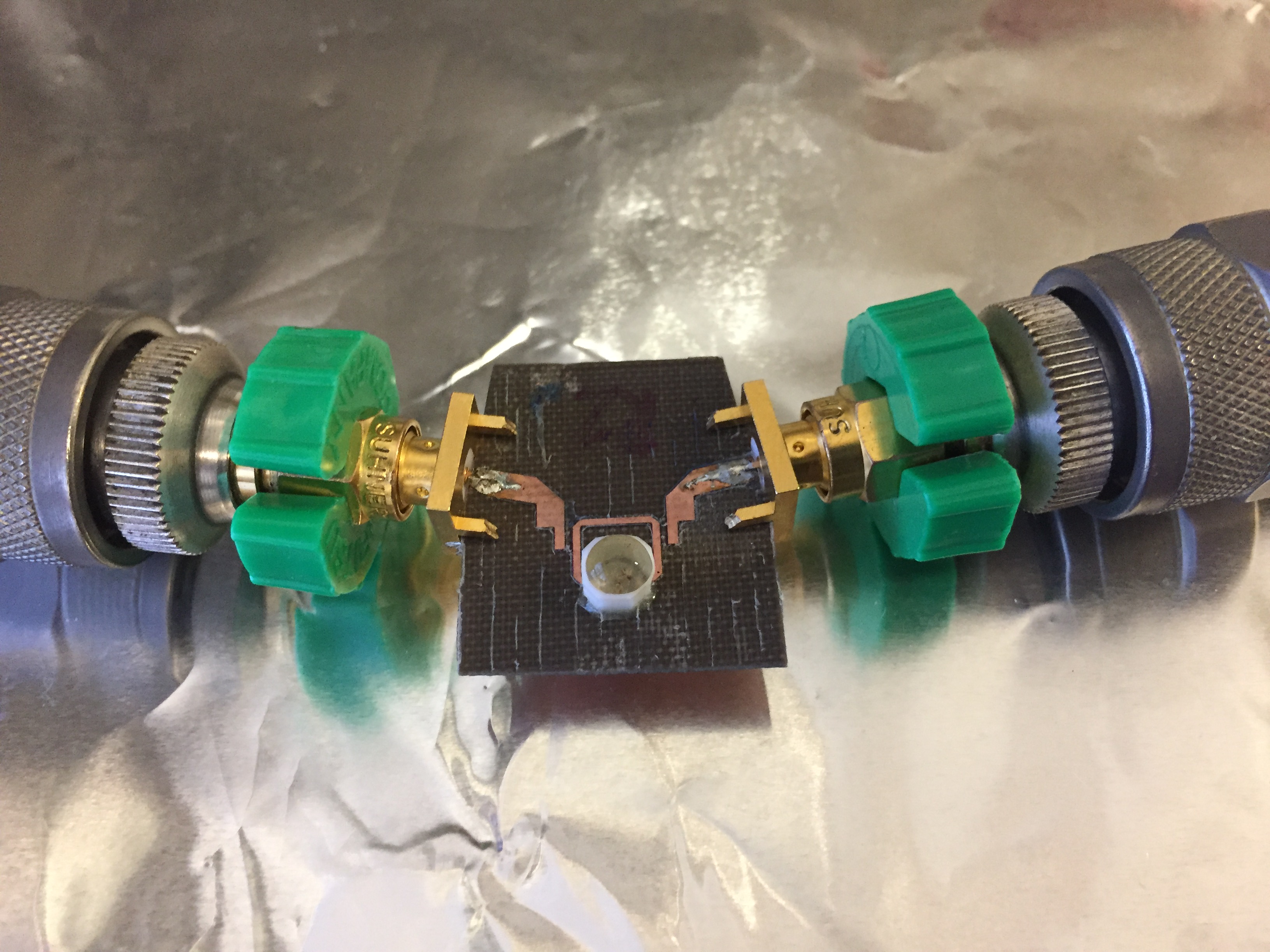

Supplement: Supplementary file 1 [file sensors-19-03779-s001.zip › Figure S2.JPG]

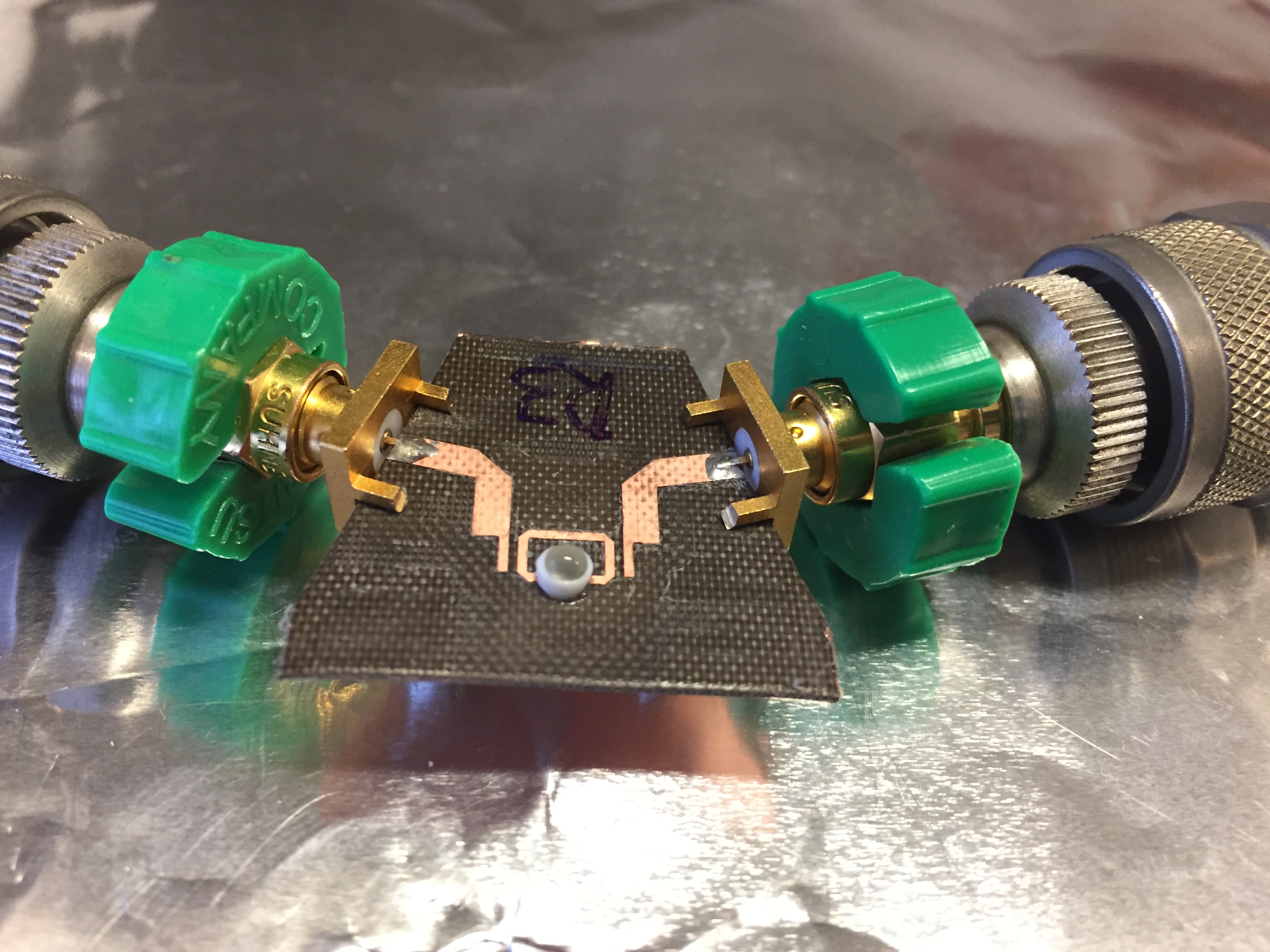

Supplement: Supplementary file 1 [file sensors-19-03779-s001.zip › Figure S3.JPG]
